# Improving Isolation of Extracellular Vesicles by Utilizing Nanomaterials

**DOI:** 10.3390/membranes12010055

**Published:** 2021-12-31

**Authors:** Haiyang Zhang, Qi Zhang, Yuanyuan Deng, Mengxi Chen, Chenxi Yang

**Affiliations:** 1 College of Pharmaceutical Sciences, Soochow University, Suzhou 215123, China; hyzhang2020@suda.edu.cn (H.Z.); 20215226089@stu.suda.edu.cn (Q.Z.); 20204226002@stu.suda.edu.cn (M.C.); 2 School of Biological Science and Medical Engineering, Southeast University, Nanjing 210096, China; 213150742@seu.edu.cn

**Keywords:** extracellular vesicles, nanomaterial-based isolation, nanomaterials

## Abstract

Extracellular vesicles (EVs) as the new form of cellular communication have been demonstrated their potential use for disease diagnosis, prognosis and treatment. EVs are vesicles with a lipid bilayer and are present in various biofluids, such as blood, saliva and urine. Therefore, EVs have emerged as one of the most appealing sources for the discovery of clinical biomarkers. However, isolation of the target EVs from different biofluids is required for the use of EVs as diagnostic and therapeutic entities in clinical settings. Owing to their unique properties and versatile functionalities, nanomaterials have been widely investigated for EV isolation with the aim to provide rapid, simple, and efficient EV enrichment. Herein, this review presents the progress of nanomaterial-based isolations for EVs over the past five years (from 2017 to 2021) and discusses the use of nanomaterials for EV isolations based on the underlying mechanism in order to offer insights into the design of nanomaterials for EV isolations.

## 1. Introduction

Enormous studies on extracellular vesicles (EVs) have advanced our knowledge of cell communication by including EVs as the new form of signaling system [1]. EVs are phospholipid bilayer-encapsulated particles that are secreted by almost all types of cells and released into extracellular environments such as blood, saliva, urine and cerebrospinal fluids. According to their biogenesis, EVs are given more specific names, including exosomes which are released during fusion of multivesicular endosomes with plasma membranes (a diameter of 30–100 nm) and microvesicles (MVs) which are directly budded from the plasma membrane (a diameter of 100–1000 nm) [2,3]. However, it is usually difficult to differentiate exosomes and MVs after their release. Therefore, “extracellular vesicle” which is suggested by the International Society of Extracellular Vesicles (ISEV) is used here to represent all the secreted vesicles [4]. As the important message carriers, EVs and their cargo, such as proteins and microRNAs, have been proven to be closely related to the pathogenesis of most types of cancer and, therefore, can serve as biomarkers for disease diagnosis, prognosis and treatment [5,6]. However, biofluids usually contain mixtures of EVs, lipoproteins and protein aggregates, among others. The exploitation of EVs as potential diagnostic and therapeutic entities thus requires methodologies that can efficiently isolate the target EVs from different biofluids.

Nanomaterials are materials with at least one dimension under 100 nm [7,8]. Because of their small size, large surface area, variable structure, and versatile functionality, scientists have applied nanomaterials to isolate EVs in the past decade in order to facilitate the development of rapid, simple, and efficient EV isolation methods [8,9]. This review outlines the advances made over the past five years (from 2017 to 2021) in nanomaterial-based isolations for EVs. It is worth mentioning that the literature on detection, analysis, and quantification of EVs was also included, with emphasis on the strategies applied for enriching or capturing EVs before detection, analysis and quantification. Because the underlying mechanism of enrichment for EVs in some of detection, analysis or quantification methods could be used for EV isolation. Here, we select some representative publications to discuss, rather than exhaustively listing all the related publications. The purpose of this review is to bring the research topic to researchers and facilitate improving the design of nanomaterials applied in EV isolations.

## 2. Conventional Isolation Approaches for Extracellular Vesicles

Although a variety of methods have emerged for EV isolation, four classes of isolation strategies are most commonly used, including ultracentrifugation (UC), size-based isolation (such as size exclusion chromatography and ultrafiltration), immunoaffinity and precipitation [10]. These conventional isolation approaches are briefly discussed here since many reviews have provided a comprehensive overview of these approaches [2,11]. Table 1 lists the advantages and disadvantages for each technique.

Up to now, the most commonly used protocol for separating EVs has involved a series of UC steps. According to their sizes and densities, the apoptotic bodies/cell debris, the MVs and exosomes are sedimented orderly with the successive increase of centrifugation forces [2]. Among the currently used EV isolation approaches, UC has been considered the “gold standard”. Due to its ease of use, UC has been widely employed in about 80% of the currently reported EV studies [11,12,13]. However, the purity of EV samples prepared by UC is often limited by the presence of co-sediment and high abundant components, such as some of non-vesicles, including protein aggregates and lipoproteins [14], which potentially compromises the subsequent EV function analysis [15]. As a result, density gradient (DG) flotation, such as the sucrose gradient or OptiPrep velocity gradient (iodixanol gradient), has been developed [16,17]. But UC-based isolations are usually extremely tedious, time-consuming and require expensive equipment [11]. Furthermore, previous studies have reported that prolonged periods of ultracentrifugation forces can detrimentally affect the structure and biological function of the isolated EVs, which would affect the downstream function studies of EVs [18].

Unlike UC, size-exclusion chromatography (SEC) as one of size-based isolations has been introduced with the most appealing feature, that is to maintain the native state of the isolated EVs [19]. When a liquid sample passes through a stationary phase with porous structures, molecules smaller than the pores of the stationary phase enter into the pores resulting in a late elution, and vice versa. Based on this principle, SEC has been widely applied to separate large molecules such as proteins and liposome particles [20,21]. Because SEC is usually operated by passive gravity flow and could elute EVs by physiological buffers, it has a minimal effect on the structure of EVs, as well as on biological function of EVs [10,22]. Because of those features, SEC is also widely used for EV isolation and several commercial SEC kits, such as qEV (IZON), are available for use. However, SEC generally results in a large volume of samples after elution, which may require an extra step to concentrate the samples. Ultrafiltration is another type of size-based isolation which separates EVs by passing them through a membrane with defined pore size or molecular weight cut-off via centrifugation or pressure. In comparison with UC, ultrafiltration is a fast EV isolation process with low equipment cost, which holds potential for industrial-scale EV preparation [2,11]. However, one of the most noticeable problems is that membranes could easily get blocked because of vesicle clogging and trapping [11,23].

Theoretically, any proteins or membrane components expressed on the membrane of EVs could be used to develop affinity-based EV isolations, such as immunoaffinity. In past decades, various EV markers have been reported including transmembrane proteins, heat shock proteins, fusion proteins (e.g., flotillins, annexins, and GTPases), lipid-related proteins, and phospholipases [24,25,26]. Among them, transmembrane proteins such as CD81, CD63, CD9, annexin, and Alix are the most widely selected marker proteins for EV isolations [27,28]. For example, several commercial EV isolation kits, such as Exosome-human CD63 isolation/detection (Invitrogen, Waltham, MA, USA) and Exosome Isolation Kit CD81/CD63 (Miltenyi Biotec, Bergisch Gladbach, Germany), were generated. Owing to isolation via interaction with specific markers, immunoaffinity-based methods are more appealing for purifying the defined subpopulations of EVs, but not for “universal” EV isolations.

Polymer-based precipitation, which is another commonly used strategy for EV isolation, decreases the solubility of EVs by creating the hydrophobic micro-environment via the interaction between the highly hydrophilic polymers and the water molecules surrounding the EVs [29]. Polyethylene glycol (PEG) is the commonly used polymer for this EV isolation [30]. Several popular commercial EV isolation kits, such as Exo-Prep (HansaBioMed, Tallinn, Estonia), Total Exosome Isolation Reagent (ThermoFisher, Waltham, MA, USA), and ExoQuick (System Biosciences, Palo Alto, CA, USA) have been developed based on PEG precipitation [11]. Generally, PEG with a molecular weight of 6000 to 20,000 Da is employed for EV precipitation since low molecular weight PEG (<5000 Da) is often utilized for the preferential hydration of proteins [30,31,32,33]. Although polymer precipitation-based EV isolation generally yields more EV samples, the obtained EV samples are typically characterized by low purity, because various water-soluble materials, including nucleic acids, lipoproteins, proteins, and even viruses, can also be precipitated by water-excluding polymers beside EVs [34,35].

As mentioned above, each isolation method has its unique advantages and disadvantages. Therefore, considerable efforts have been devoted to improving these isolation methods and details can be found elsewhere [2,11,36]. For example, dithiothreitol (DTT) or 3-[(3-cholamidopropyl)dimethylammonio]-1-propanesulfonic (CHAPS) was added into the crude exosome pellets yielded by UC. This can prevent uromodulin in urine by forming a polymeric network to trap exosomes and, thus, increase the yield of exosomes [37,38]. A simple filtration step is usually added before the UC steps in order to reduce the processing duration by concentrating samples [39]. A multiple-cycle polymeric EV precipitation or combined use with UC or SEC, was reported to improve the purity of the EV samples obtained by precipitation [40,41,42]. Moreover, UC and filtration can be used as the final step to concentrate the SEC eluent because SEC always generates a large volume of sample [43]. Despite those efforts, improvements on EV isolations are limited by either modifying isolation procedures or combining the use of those existing methods. Isolation methods for EVs could be further improved by integration with advanced nanomaterials, which will be discussed in detail in Section 3.

## 3. Nanomaterials Applied in the Isolation of Extracellular Vesicles

**Figure 1:**
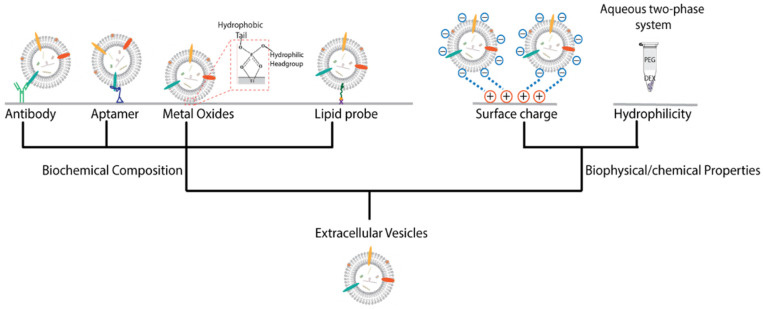
Isolation based on the surface characteristics of EVs. The EVs can be isolated by various affinity interactions including antibodies and aptamers for marker proteins, metal oxides for hydrophilic phosphate heads of phospholipids and lipid probes for lipids on the membrane of EVs. Isolation also can be performed via the biophysical or chemical properties of EV membranes, such as surface charge and hydrophilicity.

The membranes of EVs present fairly distinctive features either from biochemical composition or biophysical/chemical properties. For example, the membranes of EVs contain specific surface molecules, such as tetraspanins (e.g., CD9, CD63, CD81), proteins related to transport and fusion (e.g., flotillin, caveolin-1), heat shock proteins (e.g., Hsp90), lipid-related proteins, and phospholipids [24,25,26]. Further, the nonfunctionalized EVs have been demonstrated to carry a net-negative surface charge due to the nature of EV surface molecules, including glycans, phospho and sulpho groups [44,45]. Those specific features provide the basis for separation of EVs from others. Therefore, we will discuss those nanomaterial-based isolations according to their enriching mechanism as summarized in Figure 1. Isolation methods according to the morphology of EVs are also covered here.

### 3.1. Isolation Based on the Interaction with the Surface Molecules of Extracellular Vesicles

#### 3.1.1. Isolation Based on the Surface Proteins of Extracellular Vesicles

As mentioned above, the bilayers of EVs contain a large amount of proteins and receptors. This feature provides an excellent opportunity to develop specially designed nanomaterials for EV isolations via immune affinity interactions. Antibodies which target the surface proteins of EVs are thus typically selected and fixed on different nanomaterials for EV isolation [9,11]. Among those materials, the immunomagnetic bead is the most representative one, as it can enrich and separate EVs by a simple external magnetic field [11]. To date, several commercial immunoaffinity magnetic beads, such as Exo-Flow™ Selective Exosome Capture (System Biosciences), are available to isolate EVs with high specificity [46]. In addition to those commercial kits, many studies have also reported to modify magnetic particles with antibodies to tetraspanins, such as CD9, CD63, and CD81 [11,47].

With the aim of enhancing the interaction with EV surface proteins, nanomaterials with a high surface area have been designed and investigated over the past five years. In addition to magnetic beads, magnetic nanowires have been demonstrated as a support for immobilizing antibodies [48]. In comparison with nanoparticles, the elongated nanowire can encapsulate large amounts of magnetic nanoparticles and provide a high surface area, which allows modifications with more EV-specific antibodies, thus improving the recovery and purity of EV isolation. Based on those features, anti-CD9, anti-CD63, and anti-CD81 antibodies have been functionalized on the magnetic nanowires via streptavidin–biotin interaction. The antibody cocktail-conjugated magnetic nanowires have effectively isolated EVs from the plasma of breast and lung cancer patients [48]. Zeolitic Imidazolate Framework-8 (ZIF-8), one of the metal organic frameworks (MOFs) which can be flexibly functionalized and has tunable pore sizes, was also investigated. Owing to their distinct structure, MOFs provide more surface area for immobilization of bio-macromolecules by increasing the surface-to-volume ratio. Therefore, Zhand et al. coated the polystyrene beads with ZIF-8 and then immobilized anti-CD81, anti-CD91, anti-EpCAM and anti-PD-L1 antibodies on the beads. These ZIF-8 coated polystyrene beads were able to detect as little as 50 EVs per 10 μm bead [49]. Further, a nanostructured graphene oxide (GO)/polydopamine (PDA) film coating was applied to the surface of the channel and the Y-shaped microposts of a microfluidic device in order to increase the surface area and, thus, antibody immobilization density. Based on this nano-interface, the microfluidic device could efficiently capture EVs and facilitate development of an ultrasensitive EV ELISA assay [50].

**Figure 2:**
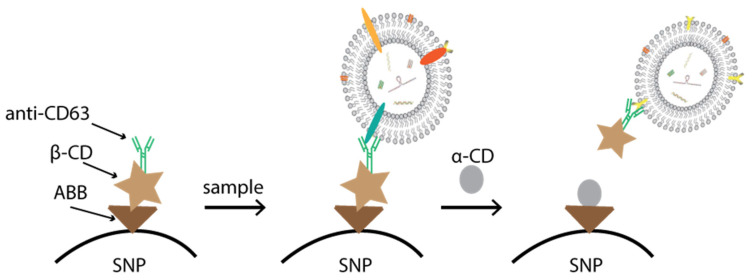
Schematic diagram for EV capture and release through the host guest interactions between β-cyclodextrin (β-CD) and 4-aminoazobenzene (AAB). The immunoaffinitive superparamagnetic nanoparticles (SNPs) are prepared by modification with AAB and then connection to β-CD-PEG_2000_-COOH. EVs in different samples are captured by immunoaffinitive SNPs and mildly eluted by adding the competitive host molecule, α-CD.

For one optimum isolation method, elution or recovery of EVs under mild condition is as crucial as EV capture for their downstream function analysis. Although immunomagnetic bead-based approaches offer a sensitive and efficient isolation method, they usually elute EVs by adding an acidic buffer or chaotropic agents. These might impair the biological activity of the EVs and cause a misleading conclusion in the subsequent function analysis [51]. To address this challenge, Cai et al. introduced host guest noncovalent interactions as smart “cleavable bridges” for EV release (Figure 2). Anti-CD63 antibodies were bound to superparamagnetic nanoparticles through host guest interactions between β-cyclodextrin (β-CD) and 4-aminoazobenzene (AAB). By eluting with the biofriendly α-CD, EVs could be released from superparamagnetic nanoparticles without impairing the biologically active substances, such as proteins and RNA, on EVs [46]. In a similar way, Kang et al. chose 3,3′-dithiobis(sulfosuccinimidylpropionate) (DTSSP) as the cleavable agent to recover EVs after capture by the anti-CD63 antibodies, which were immobilized in the inner surface of a microfluidic device. DTSSP contains a disulfide bond at the middle of the identical arm of the amine-reactive N-hydroxysulfosuccinimide ester. It can be reduced by a reducing agent, such as dithiothreitol (DTT). DTT is a water-soluble reducing reagent and is commonly used in biochemical studies, which makes it suitable for elution of EVs, because EVs are known to be more stable against changes in chemical or thermal environments [52]. Recently, a core-shell nanofiber coated with gelatin was also fabricated. Anti-CD63 antibodies were immobilized to gelatin. The elution of EVs can be achieved by incubation in water at 37 °C since gelatin is able to dissolve in water [53].

In addition to the classic antigen antibody interaction, aptamers represent an alternative approach for antibodies due to their high binding affinity, low/no immunogenicity, ease of synthesis and accessibility for different chemical modifications [8]. Aptamers, also called chemical antibodies, are RNA or single-stranded DNA (ssDNA) molecules and bind to their targets in a manner similar to antibodies. Until now, many studies have conjugated aptamers to various nanomaterials for EV isolations (Figure 1) [8,11]. Yoshida et al. coated peptide aptamers for EpCAM on the silica or polystyrene beads after being functionalized with zwitterionic MPC (2-methacryloyloxyethyl phosphorylcholine) polymers. Zwitterionic MPC polymers can reduce the non-specific binding of proteins onto a material’s surface [54]. Additionally, aptamers specific to CD63 were also reported to be absorbed onto the surface of single-walled carbon nanotubes in a microfluidic device for EV isolation before the colorimetric detection [55]. Aptamers are known to specifically bind to their targets by forming a tertiary structure which can be re-modulated by modifying the buffer system and ions (e.g., Mg^2+^ and K^2+^) responsible for the tertiary structure. This feature provides the possibility to elute EVs from aptamers under mild conditions, thereby preserving the native state of EVs [11,56]. Song et al. immobilized two DNA aptamers with high affinity and specific to CD63 proteins onto the magnetic beads. They found that CD63 aptamers not only presented a comparable diagnostic efficacy for CD63-positive breast cancer with commercial antibodies but also could release the captured EVs via a simple 0.5 M NaCl elution step [57].

To satisfy enriching specific types of EVs, proteins on the surface of EVs, except tetraspanins can be chosen as the alternative targets for EV isolation. Qi et al. chose transferrins as the capture agent for EVs due to their low immunogenicity and the abundance of transferrin receptors expressed in blood EVs. Superparamagnetic nanoparticles were labeled with transferrin and bound to the transferrin receptor on the blood EVs through transferrin transferrin receptor interaction. Results have shown that these nanoparticles have little influence on EVs and can be used in both in vitro and in vivo studies [58]. Further, the small molecule TG97, which recognizes prostate-specific membrane antigen (PSMA), was also coated onto a silica nanostructure and used to isolate EVs with expression of PSMA [59].

#### 3.1.2. Isolation Based on the Surface Lipids of Extracellular Vesicles

Complementary to characteristic surface proteins, phospholipids which constitute the foundation of EV membranes can be considered as the “universal” markers for EV isolation. Therefore, targeting the surface phospholipids has emerged as an alternative approach to isolate EVs from various biological samples [60,61,62]. For example, Tim4 proteins and annexin V, which specifically bind to the phosphatidylserine (PS) displayed on the surface of EVs, were reported to be immobilized on magnetic beads or functionalized on the surface of a microfluidic device for EV isolation [60,61]. Biotinylated annexin V and cholera toxin B chain (CTB) were also investigated to facilitate binding between EVs and streptavidin-coated magnetic nanoparticles via their affinity for phospholipids [62].

Instead of targeting one specific class of phospholipids, more efforts have emphasized enriching EVs through interaction with the “universal” phospholipids on the surface of EVs [63,64,65,66,67]. A significant feature of EVs is their uniquely curved lipid surface in the extracellular space, since highly curved membranes are commonly found to organize and compartmentalize organelles within cells, such as the Golgi [68]. By utilizing selective binding for highly curved membranes, membrane-sensing proteins, such as bradykinin, have become convenient, easy-to-synthesize novel molecular probes for targeting EVs. Gori et al. synthesized a short amino acid sequence derived from bradykinin and immobilized it on chips through chemoselective click-type reaction. It showed a higher binding capacity for EVs than anti-tetraspanins antibodies and demonstrated a potential use for EV isolations [64].

It is well known that the phospholipids in the membranes of EVs are amphiphilic with hydrophobic tails inside and hydrophilic phosphate heads outside the surface [68]. Due to this feature, metal oxides have become the method of choice for developing new EV isolation materials since some metal oxides can reversibly bind with the phosphate group and have been widely used for enrichment of phosphopeptides and water-soluble organic phosphates, among others [63,69]. Recently, Gao et al. enriched EVs with TiO_2_ microspheres. The TiO_2_-based isolation can enrich serum EVs with an isolation efficiency of 93.4% [63]. TiO_2_ microspheres have also been investigated to isolate urine EVs when combined with ultrafiltration [65]. Further, magnetic materials coated with TiO_2_ allow rapid and simple isolation with external magnetic fields. Pang et al. used Fe_3_O_4_@TiO_2_ nanoparticles to enrich and separate EVs from cell medium within 5 min and achieved a capture efficiency of 96.5% [66]. In addition to TiO_2_, Geng et al. synthesized CaZrO_3_:Sm nanosheets with a high specific surface area to bind to phosphate groups for EV isolation. In comparison with commercial TiO_2_, CaZrO_3_:Sm yielded a higher enrichment efficiency for CD63 and TSG101 proteins after analysis of the isolated EV samples [67]. Moreover, Jiao et al. synthesized Ti^4+^-modified magnetic graphene-oxide composites (GFST) and achieved tandem enrichment of EVs and EV phosphopeptides using one material. Metal or metal oxide-based materials can bind to phosphate group either from the surface phospholipids of EVs or the phosphoproteins inside EVs. Accordingly, EVs were firstly enriched by GFST from human serum and directly lysed to release the EV phosphoproteins. After digestion, GFST and the captured EV phosphopeptides were separated by a magnet. GFST performed excellently in both EV isolation and phosphopeptide enrichment. An enrichment efficiency of 83.1% was reached for EV isolation and 530 phosphoproteins were identified in serum EVs [70].

**Figure 3:**
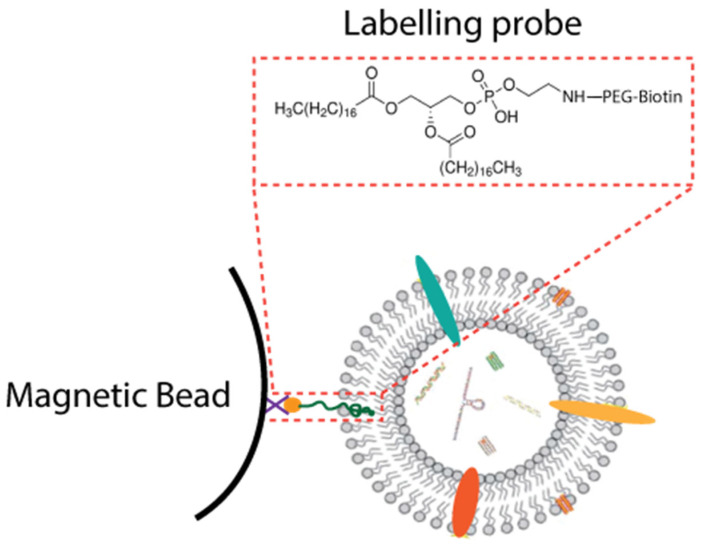
Structure of the lipid nanoprobe.

Alternatively, DSPE (1,2-distearoyl-snglycero-3-phosphethanolamine), which bear two hydrophobic fatty acid tails, has been utilized to synthesize a lipid nanoprobe for the rapid isolation of EVs from cell-culture supernatant and plasma (Figure 3). DSPE can be inserted into EV membranes via non-covalent interactions between the two hydrophobic fatty acid tails of DSPE and the lipid membranes of EVs. After being labeled with the biotin tag-lipid nanoprobes, EVs were captured by NeutrAvidin-coated magnetic particles for subsequent extraction within 15 min [71]. Wan et al. further immobilized this novel lipid nanoprobe on the nanostructure silica platform to perform EV isolation on a microfluidic device [72]. Further, the bifunctionalized magnetic beads immobilized both DSPE and Ti(IV) ions were also designed to enhance enrichment efficiency. In this design, this bifunctionalized materials can simultaneously be inserted into the EV membranes via DSPE and chelate to the phosphate group via Ti(IV) ions. It can efficiently isolate urine EVs within 1 h, with 80% recovery [73]. Moreover, hydrophilic and aromatic lipophilic groups which have high affinity toward lipid-coated EVs were utilized to isolate EVs by immobilizing them on magnetic beads [74].

#### 3.1.3. Isolation Based on the Charge and Hydrophilicity of Extracellular Vesicles

The analysis of zeta potential revealed that EVs carry a negative charge that allows positively charged molecules to capture EVs via electrostatic interactions. Consequently, anion-exchange chromatography, charge-based precipitation, and commercially available cationic particles have been explored for the possible use in EV purification (Figure 1) [46,47]. Recently, a material, called ExoCAS-2, which contains polycationic polymer-functionalized magnetic beads, was designed for EV isolations. By adjusting the salt concentration in the buffer, ExoCAS-2 could achieve capture and release EVs within about 30 min [75]. A microchip modified with chitosan was fabricated to isolate EVs as the surface charge of chitosan can be switched by simply adjusting the pH of the surrounding environment; thus, the capture and release of EVs can be achieved by using buffers with different pH. Using this microchip, EVs can be extracted from trace samples (10 μL) with a relative purity of over 90% and an 84% RNA recovery ratio within 15 min, which is impossible for traditional UC-based methods [76].

Additionally, high performance liquid chromatography (HPLC) columns integrated with new nanomaterials also provide a new strategy to isolate EVs. It is worth noting that EV samples could easily block most of the common LC columns since EVs are generally larger than the pores of the stationary phases, such as porous silica beads. Poly(ethylene terephthalate) (PET) capillary-channeled polymer (C-CP) fibers were thus introduced as stationary phases in hydrophobic interaction chromatography (HIC) workflows for EV isolation [77]. The C-CP fibers consist of an eight-legged periphery and thereby form 1 to 4 μm-wide channels, which are suitable for separating EVs. When operating in HIC mode, C-CP fibers are able to elute EVs based on their hydrophobicity via an inverse salt gradient. Compared to the organic solvents employed in the reverse phase, salt containing buffer is much better for maintaining the biological activity of EVs. Taking advantage of this, C-CP fibers have expanded their application in solid-phase extraction workflows, rather than HPLC processing platforms, by being packed into a spin-down column or micropipette tip [77,78,79,80,81].

**Figure 4:**
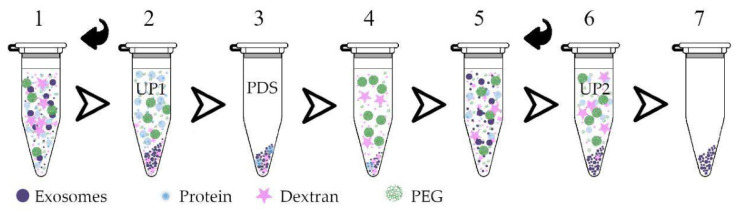
The workflow of an aqueous two-phase system (ATPS)-based EV isolation. Dextran (DEX) and PEG were added into plasma. After mixing and centrifugation, two phases formed with the upper phase (UP1) containing PEG and proteins. After removing UP1, a protein-depleting solution (PDS) was left and then mixed with PEG/DEX again. The lower phase containing DEX and EVs was collected after mixing, centrifugation and removing the PEG-rich phase again. This figure was adopted from reference [82].

Apart from isolation on the solid supports, an aqueous two-phase system (ATPS) was recently adapted to isolate EVs from urine and plasma [82,83]. ATPSs have been used to fractionate cells into subpopulations mainly according to their charge and hydrophobic surface properties [84,85,86]. Generally, small molecules prefer to be distributed between the phases while particles partition into one phase [86]. Among many hydrophilic polymers, the PEG dextran (DEX) two-phase polymer system has shown its ability to isolate EVs. EVs are usually quickly distributed into the DEX phase since the DEX phase is more hydrophilic than the PEG phase (Figure 4). An optimized ATPS could recover ~100% of EVs from urine, whereas UC only recovered 21% [82,83]. Benefiting from this simple process, Han et al. applied the PEG/DEX ATPS in a microfluidic device. This device can facilitate continuous EV isolation with 83.4% recovery efficiency without complex external equipment [87]. Seo et al. further improved ATPS by introducing an additional oil phase between the inner ATPS droplets and achieved separation of EV particles in a single DEX-rich droplet [88].

### 3.2. Separation Based on Precipitation and Size of Extracellular Vesicles

**Figure 5:**
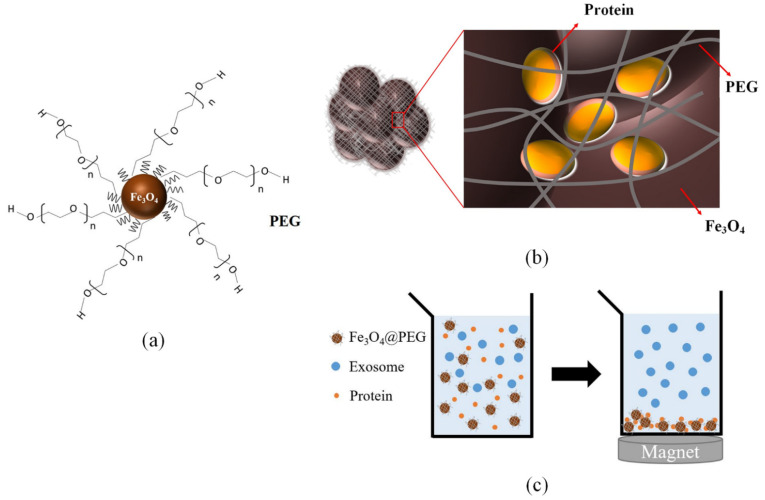
EV isolations by PEG-coated magnetic nanoparticles. (**a**) The structure of magnetic nanoparticles modified with branched PEG (**b**) Protein trapped by the reticular structures of PEG and the holes formed by agglomerates of magnetic nanoparticles (**c**) Proteins are removed by magnet. This figure was adopted from reference [90].

As mentioned in Section 2, PEG is widely employed to isolate EVs as the precipitating reagent. The PEG-based isolation approach was recently further improved by a magnetic bead-mediated selective adsorption strategy (called “MagExo”) [89]. Fang et al. found that EVs could selectively precipitate on the surface of magnetic beads under a PEG concentration of 1% to 5%, while most of the proteins remained in the supernatant. Therefore, both PEG and the magnetic beads were added into the plasma or cell-culture medium together. EVs were then absorbed onto the surface of magnetic beads and eluted by PBS. One of possible explanation for this phenomenon is the strong hydrophilic effect of PEG. Under the effect of PEG, a large number of water molecules in the solution are locked up. Consequently, the dispersion stability of EVs in the solution is changed, and they are forced to aggregate together in priority. Because magnetic beads have an abundant surface area, unstable EVs tend to aggregate on the surface of magnetic beads; thus, EVs could easily be isolated from biological fluids by a simple magnetic separation instead of a low-speed centrifuge recovery [89]. Instead of adding magnetic particles into PEG solutions, PEG-coated magnetic nanoparticles were also explored. The branched PEGs were immobilized on magnetic nanoparticles and resulted in reticular structures as shown in Figure 5. Moreover, magnetic nanoparticles are able to create a large number of holes via the formation of their agglomerates. Further results revealed that the reticular structures of PEG and the holes formed by agglomerates can trap proteins and tiny impurities. Therefore, PEG-coated magnetic nanoparticles offer an alternative strategy to isolate EVs by removing proteins and other impurities. This can reduce about 60% of proteins in fetal bovine serum without damaging the EVs [90].

Based on the principle of size-exclusion, microfluidic devices incorporated with different membranes were designed for EV isolations [11,91]. For example, two polycarbonate membranes with pore sizes of 200 and 300 nm, respectively, were separately assembled into different layers of a device. The integrated double-filtration microfluidic device can successfully enrich EVs with a size range of 30–200 nm from urine and has been applied to the study of bladder cancer [92]. Moreover, hydrogels, or forming a hydrogel-like structure, can either exclude EVs or entrap EVs according to the size of their structure and, thereby, achieve EV isolation [93,94]. For instance, super absorbent polymer (SAP) is one hydrogel which has been used as an alternative to filtration for concentrating microorganisms [95]. Due to the huge size difference between the water channel of SAP beads and EVs, relatively small molecules would be absorbed by SAP beads while EVs would be excluded and thereby concentrated [93]. Unlike SAPs, mannuronate guluronate polymer (MGP) was used to create a new EV extraction method by entrapping EVs. MGP is able to create a hydrogel structure by incorporating calcium ions. The MGP-based method can effectively isolate EVs from extremely diluted samples and avoid co-precipitating plasma proteins [94].

## 4. Conclusions

Applying nanomaterials to EV isolation methods can speed up the development of isolation methods for EVs in a simple, rapid, efficient and inexpensive way as demonstrated in this review. Powered by the advantages of nanomaterials, the application of nanomaterials goes beyond the EV isolations [8,96]. For instance, nanomaterials have been reported to facilitate the isolation of EV cargo components [97,98,99]. Recently, magnetic beads coated with complementary oligonucleotides were synthesized to enrich EV-associated microRNAs [97]. Magnetic hydrophilic materials have also demonstrated their enrichment capability for EV glycoproteins [98]. Moreover, the thermosensitive soluble polymers have been designed for the facile enrichment of EV N-glycoprotein in mild conditions [99]. Apart from the enrichment of EV cargo components, nanomaterials have been widely investigated for EV-based therapy [100]. For example, iron oxide-based nanoparticles have been employed for improving the production of EVs from stem cells in order to satisfy the needs of therapy [101].

These applications have indicated the crucial role of nanomaterials for the characterization and application of EVs, which is usually achieved by using an optimized EV isolation as a basis. Owing to the development of nanomaterials, there has been a new trend to integrate nanomaterials into conventional isolation approaches for EVs. Isolation integrated with nanomaterials can further improve EV enrichment via providing a simple isolation process, synthesizing functionalized supports/platforms, or developing a new capture agent. However, we would still like to stress that addressing the challenges faced by conventional EV isolation methods, such as the purity of the isolated EV samples, also urgently requires nanomaterial-based EV isolation [4]. It is believed that advances in nanomaterial-based EV isolation would greatly promote understanding of EV biology and facilitate translating the application of EVs in clinical settings.

## Figures and Tables

**Table 1:** Summary of the commonly used EV isolation methods.

Technique	Principle	Advantages	Disadvantages
Ultracentrifugation	Particles with different sizes and densities have different sedimentation rates during ultracentrifugation	Ease of use High purity Suitable for large volume preparation	Extremely tedious Time-consuming Low recovery High equipment cost Possible structure damage
Size exclusion chromatography	EVs pass through a porous stationary phase in which small particles enter into the pores resulting in the late elution	Maintain the native state of EVs High purity	Results in large volume of eluted samples
Ultrafiltration	EVs pass through a membrane with defined pore size or molecular weight cut-off	Fast isolation process Low equipment cost	Vesicle clogging and trapping
Immunoaffinity	Based on specific binding between surface marker proteins of EVs and immobilized antibodies	High purity and selectivity	High-cost antibodies Elution may damage native EV structure
Precipitation	Polymers decrease the solubility of EVs by creating the hydrophobic micro-environment	Ease of use High yield	Low purity Polymers affect downstream MS analysis

## Data Availability

Not applicable.
